# Understanding the relationship between asthma and autism spectrum disorder: a population-based family and twin study

**DOI:** 10.1017/S0033291721005158

**Published:** 2023-05

**Authors:** Tong Gong, Cecilia Lundholm, Sebastian Lundström, Ralf Kuja-Halkola, Mark J. Taylor, Catarina Almqvist

**Affiliations:** 1 Department of Medical Epidemiology and Biostatistics, Karolinska Institutet, Stockholm, Sweden; 2 Centre for Ethics, Lawand Mental Health (CELAM), University of Gothenburg, Gothenburg, Sweden; 3 Gillberg Neuropsychiatry Centre, University of Gothenburg, Gothenburg, Sweden; 4 Pediatric Allergy and Pulmonology Unit at Astrid Lindgren Children's Hospital, Karolinska University Hospital, Stockholm, Sweden

**Keywords:** Asthma, autism spectrum disorder, co-aggregation, LDSC, shared genetics, siblings, twins

## Abstract

**Background:**

There is some evidence that autism spectrum disorder (ASD) frequently co-occurs with immune-mediated conditions including asthma. We aimed to explore the familial co-aggregation of ASD and asthma using different genetically informed designs.

**Methods:**

We first examined familial co-aggregation of asthma and ASD in individuals born in Sweden from 1992 to 2007 (*n* = 1 569 944), including their full- and half-siblings (*n* = 1 704 388 and 356 544 pairs) and full cousins (*n* = 3 921 890 pairs), identified using Swedish register data. We then applied quantitative genetic modeling to siblings (*n* = 620 994 pairs) and twins who participated in the Child and Adolescent Twin Study in Sweden (*n* = 15 963 pairs) to estimate the contribution of genetic and environmental factors to the co-aggregation. Finally, we estimated genetic correlations between traits using linkage disequilibrium score regression (LDSC).

**Results:**

We observed a within-individual association [adjusted odds ratio (OR) 1.33, 95% confidence interval (CI) 1.28–1.37] and familial co-aggregation between asthma and ASD, and the magnitude of the associations decreased as the degree of relatedness decreased (full-siblings: OR 1.44, 95% CI 1.38–1.50, maternal half-siblings: OR 1.28, 95% CI 1.18–1.39, paternal half-siblings: OR 1.05, 95% CI 0.96–1.15, full cousins: OR 1.06, 95% CI 1.03–1.09), suggesting shared familial liability. Quantitative genetic models estimated statistically significant genetic correlations between ASD traits and asthma. Using the LDSC approach, we did not find statistically significant genetic correlations between asthma and ASD (coefficients between −0.09 and 0.12).

**Conclusions:**

Using different genetically informed designs, we found some evidence of familial co-aggregation between asthma and ASD, suggesting the weak association between these disorders was influenced by shared genetics.

## Introduction

Autism spectrum disorder (ASD) is among the most prevalent neurodevelopmental disorders worldwide, with a strong genetic basis (Sandin et al., 2017). Based on a recent meta-analysis on ASD in twins the narrow-sense heritability is estimated around 64–91% (Tick, Bolton, Happé, Rutter, & Rijsdijk, 2016). At the molecular genetic level, there has been some success in identifying rare inherited mutations and structural rearrangement (Doan et al., 2019; Iossifov et al., 2014; Woodbury-Smith & Scherer, 2018), as well as a recent genome-wide association study (GWAS) which identified five common variants robustly associated with ASD (Grove et al., 2019). Environmental risk factors associated with ASD include advanced parental age (Frans et al., 2013), maternal infections (Zerbo et al., 2015a, 2015b), nutritional deficiencies (Levine et al., 2018), exposure to air pollution or pesticides (Chun, Leung, Wen, McDonald, & Shin, 2020; von Ehrenstein et al., 2019), as well as other medical conditions during pregnancy and delivery (Lyall, Schmidt, & Hertz-Picciotto, 2014), but most studies remain inconclusive regarding potential causal interpretations.

ASD starts in early childhood and can be accompanied by both somatic and psychiatric conditions (Baxter et al., 2015). A growing body of clinical literature has pointed toward a role for the immune system in a subset of individuals with ASD (Rossignol & Frye, 2012; Xu et al., 2018; Zerbo et al., 2015a, 2015b). For example, maternal immune activation defined by having a history of immune or autoimmune diseases has been associated with ASD symptom severity in children with ASD (Patel et al., 2018). Animal models have also been able to provide some evidence on maternal immune activation and increased risk of offspring ASD (Lombardo et al., 2018). In addition, positive associations between clinical diagnoses of ASD and common allergies were reported in cross-sectional studies of children in the U.S. (Croen et al., 2019; Xu et al., 2018). Many individuals with ASD reported in clinical studies also showed hallmarks of immune-mediated diseases including cytokine abnormality and genetic mutations affecting immune function (Rossignol & Frye, 2012). However, whether the common etiology between immune-mediated diseases and ASD is due to shared genetics or environmental triggers remains unknown.

Asthma, as one of the most common immune-mediated diseases with a complex etiology and a childhood onset, has been linked to ASD in several studies, (Kotey, Ertel, & Whitcomb, 2014; Xu et al., 2018) but not all (Jonsdottir & Lang, 2017; Zerbo et al., 2015a, 2015b; Zheng et al., 2016). Familial aggregation of either condition has been reported (Sandin et al., 2014; Thomsen, van der Sluis, Kyvik, Skytthe, & Backer, 2010). Common environmental factors such as maternal medical conditions during pregnancy, parental age, and exposure to air pollution have been associated with both conditions (Gehring et al., 2015; Gómez Real et al., 2018; Mirzakhani et al., 2019; Modabbernia, Velthorst, & Reichenberg, 2017; Pagalan et al., 2019). However, very few have estimated the extent of familial co-aggregation between the two disorders (i.e. clustering of two traits among family members) and the relative contribution of genetic and environmental factors to the association between ASD and asthma. Although strong genetic correlations have been noted between asthma and other neurodevelopmental (i.e. ADHD) and affective disorders using the linkage disequilibrium score regression (LDSC) method in a genome-wide cross-trait association study, the reported genetic overlap between asthma and ASD seemed to be null (Zhu et al., 2019). Three possible reasons could be (a) limited sample size included in the ASD GWAS with 6179 and 7377 cases and controls (Cross-Disorder Group of the Psychiatric Genomics Consortium, 2013), and/or (b) a larger proportion of adult-onset asthma with more contributions from the non-genetic risk factors and exclusion of young adult-onset asthma cases in the UK Biobank sample (Pividori, Schoettler, Nicolae, Ober, & Im, 2019) (Ferreira et al., 2019) for asthma GWAS, and/or (c) that the genetic overlap estimates based on common variants and de novo mutations or structural variants were not considered. Therefore, findings from both population-based and family-based designs with sufficient sample size and minimal ascertainment bias as well as from quantitative genetic and molecular genetic designs are needed to disentangle the potential role of shared genetics or environment in the co-occurrence of ASD and asthma.

In this study, we used three different genetically informed approaches to investigate the association between asthma and ASD, since we hypothesized that some shared genetic role might explain the association. Firstly, we aimed to investigate the familial co-aggregation between asthma and ASD at the population level using data from the Swedish national registers and to what extent it is modified by the degree of genetic relatedness. Secondly, we aimed to estimate the relative contributions of genetic and environmental factors to the co-occurrence of the two disorders among twins and siblings. Thirdly, we aimed to estimate the shared common genetics of asthma and ASD by conducting post-GWAS genome-wide genetic correlation analysis.

## Material and methods

### Study population and data sources

To address the first aim, we applied a cohort design including over 1.5 million Swedish children and their siblings and cousins using data linkage between various Swedish registers via the de-identified unique personal identity number. Detailed information on each register and relevant information extracted from the register for the study is described in the supplementary material (online Supplementary Table S1). In short, we linked all children (referred to as index persons, *n* = 1 569 944) born during 1992–2007 in Sweden with their full-siblings, half-siblings, and full cousins through the Multi-Generation Register. Their asthma, ASD, migration and death information was extracted from the National Patient Register (NPR), the Prescribed Drug Register (PDR), the Total Population Register, and the Cause of Death Register. To address the second aim, we applied quantitative genetic modeling by including 620 994 eligible sibling pairs identified from the registers above and 15 963 twin pairs born during 1992–2008 whose parents responded to a telephone interview on their children's somatic and mental health at age of 9/12 years from the ongoing Child and Adolescent Twin Study in Sweden (CATSS) (Anckarsater et al., 2011). To address the third aim, we downloaded publicly available summary statistics from the GABRIEL consortium (Moffatt et al., 2010), the UK Biobank (Ferreira et al., 2019), and two recent meta-analyses (Demenais et al., 2018; Shrine et al., 2019), for asthma-relevant traits; the psychiatric genome consortium (PGC) (Cross-Disorder Group of the Psychiatric Genomics Consortium, 2013), a subset of the Avon Longitudinal Study of Children (Massrali et al., 2019), and a recent meta-analysis (Grove et al., 2019) for ASD-relevant traits (online Supplementary Table S2 for detailed information).

### Measures of asthma and ASD

Asthma was defined as individuals with (1) ⩾1 asthma-related diagnosis from NPR by 31 December 2013 according to the International Classification of Diseases, 9th or 10th revision (ICD-9/10) diagnostic codes (493, J45–J46 respectively) (World Health Organization, 1978, 2004); or (2a) ⩾2 dispensations of asthma-relevant medications including inhaled corticosteroids (ICS), leukotriene receptor antagonists (LTRA), or the fixed-dose combination of inhaled corticosteroids and long-acting *β*2 agonists independent on time of dispensations; or (2b) ⩾3 dispensations of ICS, LTRA, *β*2 agonists, or the fixed-dose combination of ICS and *β*2 agonists within a 12-month period, according to a previous validation study (Ortqvist et al., 2013). Additionally, we defined asthma if the first diagnosis or at least two or three relevant medication dispenses happened after 4.5 years of age (referred to as asthma 4.5 yr +) as a secondary outcome to exclude children who might have had transient wheezing.

For the CATSS sample, we also retrieved information on parental-reported asthma ever based on one of the core International Study of Asthma and Allergies in Childhood questions ‘Does he/she have or ever have had asthma?’ from the telephone interview.

ASD ever was defined as children with at least one ASD-related inpatient and outpatient diagnosis from the NPR since birth to December 31, 2013 (later referred as ASD ever) according to the ICD-9/10 codes (299A and F84.0, F84.1, F84.5, F84.9) (Frans et al., 2013; Lundstrom, Reichenberg, Anckarsater, Lichtenstein, & Gillberg, 2015). We also retrieved ASD diagnostic subtypes for autistic disorder (F84.0) and Asperger's syndrome (F84.5) as secondary outcomes (Idring et al., 2012).

For the CATSS sample, we retrieved information on each twin's ASD traits based on the Autism-Tics, ADHD, and other Comorbidities inventory, an open-access and comprehensive tool for screening childhood ASD and other targeted disorders based on DSM-IV criteria (Hansson et al., 2005). ASD traits were assessed using the sum of scores from 17 symptom-based questions reported by their parents, which has been validated with good to excellent sensitivity and specificity (Marland et al., 2017). We assumed this would cover the full range of manifestations of the putative quantitative traits in addition to the clinical diagnostic data.

### Statistical analyses

#### Familial co-aggregation of asthma and ASD

Prior to investigating the familial co-aggregation between ASD and asthma, we explored the comorbidity by estimating the within-individual risk of asthma/ASD and reported odds ratios (ORs) with 95% confidence intervals (CI) of ASD with asthma compared to those without asthma using logistic regression. We excluded individuals who had migrated or died before age of 6. Then, we estimated the familial co-aggregation of asthma and ASD and reported ORs of ASD in relatives with different degrees of relatedness (i.e. full-siblings, maternal and paternal half-siblings, and full-cousins) to index persons with asthma compared to relatives of index persons without asthma using logistic regression. Estimates were reported from (1) crude models, (2) models adjusted for potential confounders, i.e. calendar year of birth, parity, sex, 3) additionally adjusted for asthma in the relatives, and (4) also adjusted for maternal and paternal age at child birth for both index person and relatives. In families with multiple sibling or cousin pairs sharing the same parents and grandparents, we kept all possible pairs for analyses, and used the sandwich estimator for standard errors to correct for familial clustering. The analyses were performed in Stata version 15 (Stata, Cary, NC, USA).

#### Quantitative genetic modelling

We performed quantitative genetic analyses separately with non-twin sibling pairs from the register linkage and with twin pairs from CATSS due to different asthma and ASD measures. For families with multiple non-twin siblings, we randomly selected one pair of siblings. Detailed information about the methods is described elsewhere (Boomsma, Busjahn, & Peltonen, 2002). In short, individual cross-trait (i.e. phenotypic) correlation, cross-relative within-trait, and cross-relative cross-trait correlations were first estimated via tetrachoric correlation for binary measures (i.e. asthma and ASD ever) and biserial correlation for an association between binary and continuous measures (i.e. parental-reported asthma and ASD traits). We then applied different univariate and bivariate quantitative genetic models to quantify the proportion of variation in liability to ASD and asthma that was due to genetic and environmental variation. For non-twins siblings, the models assume that dichotomous traits are underpinned by a normally distributed liability for asthma and ASD ever (i.e. liability-threshold model). For twins from CATSS, the models predict the expected mean and variance for parental-reported ASD trait, and assume parental-reported asthma modeled according to the liability-threshold model. We estimated the fraction of variance in liability explained by (1) additive genetic effects [i.e. narrow-sense heritability (noted as A), assuming monozygotic (MZ) twins sharing 100%, dizygotic (DZ) twins and full-siblings sharing 50%, and half-siblings sharing 25% of their aggregated genes], (2a) non-additive/dominant genetic effects (noted as D, assuming MZ twins sharing 100%, DZ twins and full-siblings sharing 25% of their dominant genes, together with A accounting for broad-sense heritability), or (2b) shared environmental effects (environmental influences which MZ, DZ, and full-siblings share 100%, maternal half-siblings are assumed to share 100%, and paternal half-siblings are assumed to share 0% (noted as C), and (3) unique environmental effects (environmental influences unique to each individual with measurement error included, noted as E). We tested univariate and bivariate ACE, ADE, AE models respectively. For simplified interpretation of bivariate ADE models, we also presented the broad-sense heritability H, i.e. (A + D). The likelihood ratio test and the Akaike information criterion (AIC) were used to select the best-fitting model. Due to the restrictions in the number of parameters that can be estimated in a model, the total variance of the observed trait was set to be one (i.e. A + (C/D) + E = 1), and the Wald method was used to calculate 95% CIs for all parameter estimates. All analyses were performed in R (version 3.6.2) with the OpenMx package (version 2.14.1) (Neale et al., 2016).

#### Molecular genetic approach using LD score regression

We investigated the SNP-level heritability, i.e. variance explained by genetic variants, and genetic correlation of asthma and ASD using LDSC analysis (Bulik-Sullivan et al., 2015). Briefly, summary-level data (e.g. *p* values, beta coefficient or OR) from GWAS were included to estimate the association between population-stratified χ^2^-statistics from GWAS and linkage disequilibrium (LD), i.e. by comparing LD scores of the SNPs. Analysis was performed using Python 2.7.5 and the robustness of the results was tested by constraining the intercept from 1 to 0.7 (to address the potential error from population stratification *v.* error from sampling noise as well as the sample overlap).

#### Ethical consideration

The study was approved by the Regional Ethics Review Board in Stockholm.

## Results

Characteristics of sibling and cousin pairs identified from the cohort of children born in Sweden from 1992 to 2007 are presented in online Supplementary Table S3. The prevalence of asthma was 11.23–14.85% for females and 15.07–19.42% for males, and for ASD were 0.72–1.58% for females and 1.82–3.49% for males. Half-siblings had higher asthma and ASD prevalence (17.16 and 2.55% for maternal half-siblings, 16.37 and 2.26% for paternal half-siblings), than full siblings (13.27 and 1.29%) and cousins (15.07 and 1.40%).

### Familial co-aggregation of asthma and ASD

**Fig. 1.**
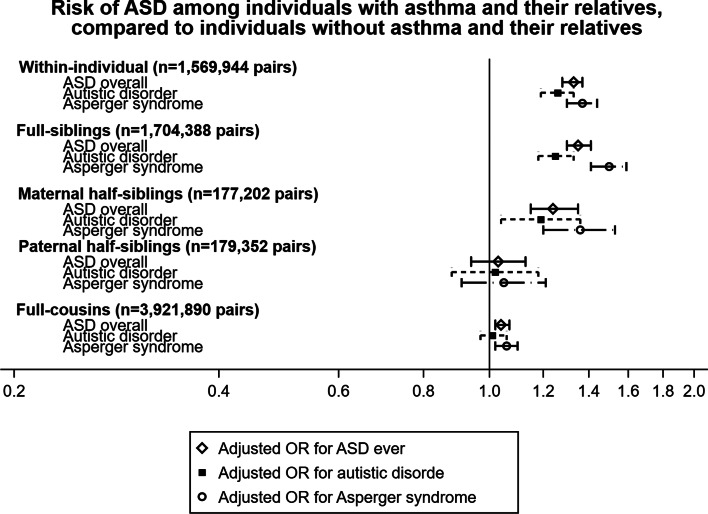
Odds ratios (ORs) of autism spectrum disorders (ASD) among individuals with asthma and their relatives, compared to individuals without asthma and their relatives. Estimates were plotted based on crude models and models adjusted for birth year, sex, and parity.

In Fig. 1, we present familial co-aggregation of asthma and ASD using sibling and cousin data. We observed an increased risk of ASD within individuals with asthma compared to those without asthma (OR 1.39, 95% CI 1.35–1.44). After controlling for birth year, sex, and parity, all relatives were at slightly higher risk for ASD, and the risk magnitude gradually decreased from full-siblings (adjusted OR 1.44, 95% CI 1.38–1.50), to maternal half-siblings (adjusted OR 1.28, 95% CI 1.18–1.39), to paternal half-siblings (adjusted OR 1.05, 95% CI 0.96–1.15) then to full-cousins (adjusted OR 1.06, 95% CI 1.03–1.07). We observed similarly increased risk for subtypes of ASD, i.e. Asperger's syndrome and autistic disorders, when adjusting for the exposure trait in the outcome person additionally (online Supplementary Table S4), and when comparing the relatives of individuals with *v.* without asthma 4.5 yr+ to exclude the transient wheezing cases (online Supplementary Table S5).

### Quantitative genetic and environmental contributions to the overlap between asthma and ASD

Table 1 reports the phenotypic, intra-class, and cross-relative cross-trait correlations between asthma and ASD. The phenotypic correlations did not exceed 0.13, and were relatively consistent for all types of relatives. Intra-class correlations for asthma and ASD decreased with decreased degrees of genetic relatedness, suggesting substantial genetic components for each trait respectively (see online Supplementary Table S6 for estimates on all ASD phenotypes). The cross-relative cross trait correlations were slightly lower with decreased degrees of genetic relatedness, suggesting weak genetic influences on the familial co-aggregation between ASD and asthma. Similar patterns were seen among sibling pairs when asthma was limited to 4.5 yr + (online Supplementary Table S7).

**Table 1.** Cross-relative within-trait, cross-trait within-individual, and cross-relative cross-trait correlations for asthma and ASD

Type of relatives	No. of concordant pairs^a^	No. of double concordant pairs^b^	Phenotypic correlation for asthma and ASD ^c^	ICC for asthma^d^	ICC for ASD	CRCT correlation for asthma and ASD^e^
MZ twins (*n* = 4842 pairs)	–	–	0.10 (0.08–0.12)	0.89 (0.88–0.90)	0.69 (0.68–0.71)	0.09 (0.07–0.11)
DZ twins (*n* = 11 244 pairs)	–	–	0.13 (0.11–0.14)	0.39 (0.38–0.41)	0.26 (0.24–0.28)	0.08 (0.07–0.09)
Full-siblings (*n* = 523 893 pairs)	2416	68	0.08 (0.08–0.08)	0.34 (0.33–0.34)	0.42 (0.42–0.43)	0.06 (0.06–0.06)
Maternal half-siblings (*n* = 50 303 pairs)	497	11	0.08 (0.07–0.08)	0.21 (0.21–0.22)	0.24 (0.23–0.25)	0.06 (0.05–0.06)
Paternal half-siblings (*n* = 46 798 pairs)	416	3	0.10 (0.09–0.10)	0.12 (0.11–0.13)	0.18 (0.17–0.19)	0.02 (0.01–0.02)

a Number of concordant pairs denotes number of sibling pairs where one sibling had asthma and the other sibling had ASD. For twins, parent-reported asthma and ASD trait was measured as scores, so concordant pairs not applicable.

b Number of double concordant pairs denotes sibling pairs where both siblings had asthma and ASD. .

c Phenotypic correlation denotes the correlation coefficient between asthma and ASD within individual for non-twins.

d Intraclass correlation (ICC) denotes the correlation between individual and his/her relative on the trait.

e Cross-relative cross-trait (CRCT) correlation denotes the tetrachoric/biserial correlation when the individual had asthma, and the relative of the individual had ASD.

Table 2 shows the additive genetic, dominant genetic, shared environmental, and unique environmental effects influencing both asthma and ASD, based on 15 963 pairs of twins and, separately, 620 994 sibling pairs from the best-fitting quantitative genetic models (see all tested models in online Supplementary Tables S8 & S9). In univariate analysis of twins, genetic effects accounted for a substantial proportion of the total variation in liability to parental-reported asthma (*A* = 0.67, 95% CI 0.53–0.82; *D* = 0.22, 95% CI 0.07–0.37) and ASD trait (*A* = 0.29, 95% CI 0.22–0.36; *D* = 0.43, 95% CI 0.36–0.50). Unique environment accounted for a relatively small proportion of the total variance in liability to asthma (*E* = 0.11, 95% CI 0.09–0.13) and ASD (*E* = 0.28, 95% CI 0.27–0.29). In univariate analysis of non-twin siblings, genetic effect accounted for 59% of the total variation for asthma (*A* = 0.59, 95% CI 0.50–0.67) and 75% of that for ASD (*A* = 0.75, 95% CI 0.55–0.95), followed by unique environment (*E* = 0.37, 95% CI 0.33–0.42 for asthma and *E* = 0.20, 95% CI 0.09–0.31) for ASD) and minimal from a shared environment. In a bivariate analysis of twins, broad-sense heritability estimates (H) were 0.89 (95% CI 0.87–0.91) and 0.50 (95% CI 0.49–0.51) for parental-reported asthma and ASD trait respectively, which the latter was slightly lower than estimates from the univariate models. The additive and dominance genetic correlations were of opposite sign, additive genetics contributed positively while dominance contributed negatively. Together A and D accounted for 89% of the correlation. The genetic correlations between parental-reported asthma and ASD traits were weak but still greater than the corresponding unique environmental correlation (*r*_A_ = 0.31, *r*_D_ = −0.13, *r*_H_ = 0.20, *v. r*_E_ = 0.03). In a bivariate analysis of non-twin siblings, additive genetic (*r*_A_ = 0.20) and shared environment (*r*_C_ = 0.03) contributed positively and unique environment (*r*_E_ = −0.14) contributed negatively to the phenotypic correlation between asthma and ASD ever.

**Table 2.** Best-fitted quantitative genetic models' estimates for asthma and ASD using Swedish twins and siblings

	Traits	Type of relatives	Number of pairs		Univariate Model			Bivariate Model											
			Within disorder		Proportion of variance within disorder explained (95% CI)			Proportion of variance within disorder explained (95% CI)				Proportion of Covariance with asthma explained (95% CI)				Correlation with Asthma (95% CI)			
			Concordant pairs^a^	Discordant pairs	A	D	E	A	D	H	E	A	D	H	E	A	D	H	E
*Twins* (*n* *=* *15 963 pairs*)	Parent-reported asthma	MZ twins	503 / 3895	416	0.67 (0.53–0.82)	0.22 (0.07–0.37)	0.11 (0.09–0.13)	0.65 (0.50–0.79)	0.24 (0.09–0.39)	0.89 (0.87–0.91)	0.11 (0.09–0.13)	1.53 (0.77–2.30)	−0.65 (−1.49 to 0.20)	0.89 (0.68–1.10)	0.11 (−0.10 to 0.32)	0.31 (0.15–0.47)	−0.13 (−0.30 to 0.05)	0.20 (0.14–0.26)	0.03 (−0.02 to 0.08)
		DZ twins	546 / 8328	2275															
	ASD trait	MZ twins	–	–	0.29 (0.22–0.36)	0.43 (0.36–0.50)	0.28 (0.27–0.29)	0.13 (0.10–0.16)	0.37 (0.33–0.41)	0.50 (0.49–0.51)	0.50 (0.49–0.51)								
		DZ twins	–	–															

					A	C	E	A		C	E	A		C	E	A		C	E
*Siblings* (*n* *=* *620 994 pairs*)	Asthma	Full-siblings	20 989 / 397 036	105 868	0.59 (0.50–0.67)	0.04 (0.00–0.09)	0.37 (0.33–0.42)	0.46 (0.40– 0.52)		0.10 (0.08– 0.13)	0.43 (0.40– 0.46)	1.49 (0.88–2.09)		0.00 (−0.24 to 0.23)	−0.49 (−0.88 to 0.08)	0.20 (0.11– 0.29)		0.00 (−0.32 to 0.31)	−0.13 (−0.21 to 0.04)
		Maternal Half-siblings	2224 / 35 361	12 718															
		Paternal Half-siblings	1613 / 33 052	12 133															
	ASD ever	Full-siblings	754 / 510 754	12 385	0.75 (0.55–0.95)	0.05 (−0.05 to 0.15)	0.20 (0.09–0.31)	0.76 (0.68– 0.84)		0.03 (0.00– 0.07)	0.21 (0.16– 0.26)								
		Maternal Half-siblings	92 / 47 933	2278															
		Paternal Half-siblings	56 / 44 765	1977															

Abbreviations: A, additive genetic component; D, non-additive/ dominant genetic component; H, broad-sense heritability component, which is A + D; C, shared environmental component; E, non-shared environmental component (including measurement errors); ASD, autism spectrum disorder.

a Concordant pairs denotes number of both affected / unaffected pairs. Not applicable for twins, as ASD is measured on a continuous scale.

### Genetic correlation between asthma and ASD from GWAS summary statistics

Table 3 presents the SNP-based heritability estimates and the genetic correlation of asthma and ASD traits using publicly available GWAS summary data. Overall, we observed low to moderate SNP-based heritability for asthma and ASD (*h*^2^_SNP_ = 0.05–0.11 and 0.10–0.46 respectively). The intercepts estimated from SNP-based heritability analyses were between 0.96 and 1.06, suggesting minimal population stratification to the inflation in χ^2^ statistics (online Supplementary Table S10). None of the genetic correlations between asthma and ASD was statistically significant (e.g. coefficients *r*_g_ ranging from −0.09 to 0.12, all *p* values ⩾ 0.05), and were rather robust based on different GWAS summary data sources.

**Table 3.** SNP-based heritability (*h*^2^_SNP_) and genetic correlation (r_g_) estimates of asthma and ASD

Trait 1	Trait 2	*h*^2^_SNP (trait 1):_*h*^2^_SNP (trait 2)_	Genetic correlation, r_g_ (s.e.)	p-value
Childhood asthma (Moffatt MF et al.)	ASD (PGC)	0.1161:0.4247	0.116(0.1095)	0.2897
	ASD (Grove J et al.)	0.1255:0.2089	−0.0948(0.0826)	0.2513
	Autistic trait (Massrali A et al.)	0.1252:0.1224	0.063(0.261)	0.8094
				
Childhood asthma (Demenais F et al.)	ASD (PGC)	0.0592:0.4268	0.1072(0.079)	0.1749
	ASD (Grove J et al.)	0.0586:0.2031	0.0073(0.0611)	0.9052
	Autistic trait (Massrali A et al.)	0.0587:0.1461	0.0205(0.1564)	0.1309
Childhood asthma (Ferreira MA et al.)	ASD (PGC)	0.0654:0.451	0.0072(0.0465)	0.8775
	ASD (Grove J et al.)	0.0659:0.2023	0.0338(0.0422)	0.4232
	Autistic trait (Massrali A et al.)	0.0671:0.0986	0.1769(0.1441)	0.2196
Self-reported asthma (UKB)	ASD (PGC)	0.0569:0.455	−0.0038(0.0498)	0.9387
	ASD (Grove J et al.)	0.0571:0.2022	0.0826(0.0427)	0.0528
	Autistic trait (Massrali A et al.)	0.0579:0.0997	−0.0272(0.1247)	0.8273
Doctor-diagnosed asthma (UKB)	ASD (PGC)	0.0675:0.455	−0.0264(0.0719)	0.7139
	ASD (Grove J et al.)	0.0683:0.2022	0.0219(0.0585)	0.7087
	Autistic trait (Massrali A et al.)	0.07:0.0997	−0.0899(0.1723)	0.6018
Moderate-to-severe asthma (Shrine N et al.)	ASD (PGC)	0.1017:0.4662	0.0484(0.0674)	0.4723
	ASD (Grove J et al.)	0.1017:0.2018	0.0352(0.0559)	0.5285
	Autistic trait (Massrali A et al.)	0.1039:0.1058	−0.0542(0.1906)	0.7761

**Abbreviations:** PGC, psychiatric genomic consortium; s.e., standard error; UKB, UK Biobank.

## Discussion

In this large population-based study using different genetically informed approaches to disentangle the familial co-aggregation of asthma and ASD, we first reported some evidence of familial co-aggregation using nearly complete and accurate case ascertainment of asthma and ASD from nation-wide records of diagnoses and prescriptions made by healthcare professionals in Sweden. Using the twin and sibling samples, we further identified latent additive and non-additive shared genetics mainly contributed to the phenotypic association of the two disorders, but not the latent shared environment factors. However, a non-significant genetic correlation based on current GWAS summary data could not confirm the genetic overlap between the two disorders at the common genetic variant level, suggesting a possible power issue or more complicated shared genetic liabilities that require further investigations.

We found a positive association between asthma and ASD, which was similar to the results observed from two large-scale cross-sectional or case−control studies (Kotey et al., 2014; Xu et al., 2018), but only partly from a meta-analysis (Zheng et al., 2016). The first two studies reported approximately 20–30% increased odds of ASD associated with respiratory allergy broadly or asthma using U.S. population-based survey samples and Health Insurance data in Taiwan (Kotey et al., 2014; Xu et al., 2018). The meta-analysis results showed asimilar estimate (OR 1.26, 95% CI 0.98–1.61) when pooling cross-sectional studies with moderate heterogeneity, but not when pooling five case−control studies (OR 0.98, 95% CI 0.68–1.43) (Zheng et al., 2016). Notably, one case−control study using a large sample of cases and controls from Kaiser Permanente that was given significantly more weight in the meta-analysis found even 20% lower odds for ASD for boys diagnosed with asthma than those without, which could possibly be due to inconsistent definitions and incomplete ascertainment of transient asthma especially among boys, or the potential difference in sample selection with regard to parental characteristics (i.e. no information on parental socioeconomic status, smoking during pregnancy, family history of asthma provided) (Zerbo et al., 2015a, 2015b). Familial co-aggregation of asthma and ASD has not been previously studied except for parental asthma and offspring ASD (Croen et al., 2019; Gong et al., 2019). We observed there was a slight decrease in the magnitude of the associations with more distant genetic relatedness after covariate adjustment, suggesting some weak evidence for shared familial liability between asthma and ASD, regardless of ASD subtypes. Furthermore, the difference among maternal and paternal half-siblings indicate maternal factors might explain the shared liability additionally. Interestingly, medical conditions of the mother, not the father, e.g. autoimmune diseases and birth-related complications have been linked to both asthma and ASD (Beasley, Semprini, & Mitchell, 2015; Croen et al., 2019; Modabbernia et al., 2017), where maternal cellular immune response (including elevated TNF-*α* and/or cytokine levels) might explain the association to some extent (Jones et al., 2017). However, further studies are needed to confirm a difference in co-aggregation between maternal and paternal half-siblings.

The previously documented comorbidity of asthma and ASD has led to our interest in investigating the familial co-aggregation of these conditions, especially testing the potential presence of a shared genetic and/or environment liability. Given a gradually decreased co-aggregation estimated from sibling and cousin analyses, the co-aggregation could be due to shared genetics. Using twins to decompose the variance, we confirmed moderate-to-high heritability of each trait (Sandin et al., 2017; Ullemar et al., 2016), and found a weak genetic correlation (i.e. *r*_A_ = 0.20 for non-twin siblings, *r*_H_ = 0.20 for twins) which partially explained the phenotypic correlation. A similar but weaker correlation for asthma and ADHD was reported in our previous studies (Mogensen, Larsson, Lundholm, & Almqvist, 2011). Also consistently with our previous findings (i.e. phenotypic correlation similar to CTCT correlation in MZ twins), the unique environment and measurement error component did not seem to account for the phenotypic correlation. Confirming the results in future studies is still needed.

Although the familial co-aggregation and the quantitative genetic analysis suggest a possible contribution from shared genetics, we did not identify genetic overlap between asthma and ASD at individual SNP variant level using publicly available summary statistics. Zhu and colleagues previously identified a genetic correlation between asthma and ADHD, anxiety, or depression, but not for autism, which was in line with our results (Zhu et al., 2019). However, several factors might contribute to this null funding. First, it is notable that reported summary data for asthma phenotypes can differ by age of onset and the source of ascertainment (i.e. self-reported, and doctor-diagnosed). In particular, childhood asthma cases only represent 5% of the total number of self-reported cases in the UK Biobank. We therefore applied two large childhood asthma GWAS summary data to replicate. Second, the sample overlap between reported summary statistics was unknown. This motivated us to test the genetic correlation at different intercept constraints, despite robust null finding (results available on request). Third, we still relied on the GWAS results even though previous findings seem to suggest common genetic variants only explain a relatively small proportion of the genetic variation for asthma or ASD liabilities (i.e. SNP-based heritability estimates being much lower than twin model-based heritability estimates). Therefore, we still cannot rule out the possibility of an underpowered LDSC analysis, which preclude us from drawing firm conclusions on familial co-aggregation of asthma and ASD. Furthermore, other variants (e.g. copy number variants, de novo mutations etc.) may still explain the shared genetic etiologies seen in the twin and sibling samples.

While parent-offspring studies have been used to examine familial co-aggregation between asthma and ASD, to our knowledge, no previous studies have demonstrated that co-aggregation exists in other types of kinship. The Swedish national registers with high data quality, large longitudinal population-based cohorts of singletons and twins, and validated measures for asthma and ASD were used to explore the familial co-aggregation. In addition, ascertainment bias due to different healthcare-seeking behavior within a family was minimal with our study design, as the comparisons were made between cases and their relatives at different levels of genetic relatedness.

We acknowledge several potential limitations of this study. First, the NPR contains almost complete diagnostic information from inpatient and outpatient care since 1987 and 2001 respectively, but asthma diagnosis made at primary healthcare centers and some private clinics were not included. The PDR would identify most asthmatic children as long as parents have dispensed some asthma medications for the child. However, we were not able to further define transient, allergic, or eosinophilic asthma, which may hinder information on a potential pathophysiological link. A further limitation was the right censoring causing a 6-year minimum follow-up in the population-based sample. However, this should still allow us to include most of the ASD cases diagnosed at an early age, according to a previous validation study (Idring et al., 2012). Second, the potential maternal effects on ASD and asthma we saw in the familial aggregation analyses could not be further investigated with the twin models, as those models are not designed for such comparisons. Third, exposure to air pollution, which can be a confounder, was not adjusted for in the models. This would possibly have biased our results on the familial co-aggregation toward the null, as air pollution would affect asthma and ASD in the same direction. Finally, continuous-scale data on other ASD phenotypes were based on parental reports which may be less reliable than clinical diagnoses, and results were limited for the sub-analyses and LDSC regression, which could be further investigated in future studies.

## Conclusions

To summarize, for the first time to our knowledge, this study provides some evidence of familial co-aggregation of asthma and ASD using population-based register data. By applying different genetically informed designs, our analysis tried to further understand the genetic overlap between asthma and ASD, which could be valuable information for clinicians and their patients. A weak shared genetic variability also seemed to explain the observed phenotypic correlation to some extent, but maybe not directly through common SNPs. However, our findings are important for raising the awareness in health care that there is an association between asthma and ASD and further investigations are needed to unravel the relationships between asthma and ASD subtypes, the interacting role of additive and dominant genetic effect, as well as the maternal factors.

## Supplementary material

For supplementary material accompanying this paper visit https://doi.org/10.1017/S0033291721005158. click here to view supplementary material
