# Supplementary material for: Understanding the relationship between asthma and autism spectrum disorder: a population-based family and twin study
Source: Psychol Med. 2022 Apr 7;53(7):3096–104. doi: 10.1017/S0033291721005158 (PMC10235668; doi:10.1017/S0033291721005158)
Supplement: Supplementary file 1 [file S0033291721005158sup001.docx]

Table S1. Data sources from national and regional registers for linkage

| **Register name** | **Register managed by** | **Register description** | **Information and time points retrieved from the register for the study** |
| --- | --- | --- | --- |
| **Medical Birth Register (MBR)** | The National Board of Health and Social Welfare | The register covers more than 99% of the birth in Sweden since 1973 (Swedish National Board of Health and Welfare, 2003). Diagnoses are coded according to the 7^th^ to 10^th^ versions of the International Classification of Diseases (ICD 7-10). Medications are either recorded in free text or as Anatomical Therapeutic Chemical (ATC) codes. | Maternal age at child birth, |
|  |  |  | Child’s gender and date of birth, |
|  |  |  | Parity |
| **National Patient Register (NPR)** |  | The register covers nationwide information from 1961 on inpatient care with complete coverage since 1987 and about 80% of the visits to outpatient specialist care since 2001 (Ludvigsson et al., 2011). Diagnoses are coded according to ICD 7-10. | Incident asthma |
|  |  |  | Incident autism spectrum disorder (ASD) |
| **Swedish Prescribed Drug Register (SPDR)** |  | The register covers around 80% of dispenses of all prescribed drugs since July 2005 (Wettermark et al., 2007). All medications are recorded in free text and ATC codes. | Incident asthma by 1) ≥2 dispensed ICS and/or LTRA and/or β2-ICS, independent on time between distribution, and/or 2) ≥3 dispensed ICS and/or LTRA and/or β2-ICS and or β2, within 12-month period, according to the validation study (4). |
| **Cause of Death Register (CDR)** |  | The register covers more than 99% death in Sweden with information on primary and secondary causes of death and date of death (Johansson & Westerling, 2000). | Identifying individuals who died during follow-up. |
| **Multi-Generation Register (MGR)** | Statistics Sweden | The register covers data of more than nine million individual, with >95% information on mothers and fathers of the index person born from 1932 and alive in 1961 (Ekbom, 2011). | Identifying biological and adoptive parents, full-siblings, half-siblings, and full-cousins |
| **Total Population Register (TPR)** |  | The register covers basic information of population composition e.g. gender, age, etc. and could be linked with other registers via unique personal identificaiton number (Ludvigsson, Otterblad-Olausson, Pettersson, & Ekbom,2009). | Paternal age at child birth, emigration and immigration records. |
| **Swedish Twin Register** | Department of Medical Epidemiology and Biostatistics, Karolinska Institute | The register included information on genetic and environmental exposures and various health outcome measures on 95,000 twin pairs living in Sweden (Magnusson et al., 2013). | Zygosity, age, sex |
|  |  |  | Parent-reported asthma and ASD traits at age of 9/12 years. |

Table S2. Detailed information of GWAS summary data of asthma and ASD

| Source | PMID | Phenotype | Sample size | Reference panel used | Statistical method used | Number of SNPs reported in the summary statistics | Summary data download URL |
| --- | --- | --- | --- | --- | --- | --- | --- |
| **Asthma** | |  |  |  |  |  |  |
| Moffat M et al. | 20860503 | Childhood-onset asthma | 26 475 | HapMap2, CEU, r21 | Logistic regression model with robust sandwich estimator | 567 589 | <https://beaune.cng.fr/gabriel/gabriel_results.zip> |
| Demenais et al. | 29273806 | Childhood-onset asthma | 127 669 | HapMap2 | Logistic regression model + fixed-effects meta analysis with inverse-variance + random-effects meta analysis with Der Simonian and Laird estimator | 2 001 282 | <ftp://ftp.ebi.ac.uk/pub/databases/gwas/summary_statistics/DemenaisF_29273806_GCST006862/TAGC_meta-analyses_results_for_asthma_risk.zip> |
| Ferriera MA et al. | 29083406 | Childhood-onset asthma | 314 633 | 1000 Genome Project | Linear mixed model + Logistic regression + inverse-variance-weighted fixed-effects meta-analysis | 9 020 834 | <https://genepi.qimr.edu.au/staff/manuelF/gwas_results/main.html> |
| UK Biobank (UKB) | Neale's lab | Self-reported asthma | 361 141 | Haplotype Reference Consortium panel + UK10K + 1000 Genomes Phase 3 | Logistic regression | NA | <https://www.dropbox.com/s/8im5taqmydndg5l/20002_1111.ldsc.imputed_v3.both_sexes.tsv.bgz?dl=1> |
|  |  | Doctor-diagnosed asthma | 91 787 |  | Logistic regression | NA | <https://www.dropbox.com/s/f717sasnggr3eei/22127.ldsc.imputed_v3.both_sexes.tsv.bgz?dl=1> |
| Shrine N et al. | 30552067 | Moderate-to-severe asthma | 57 695 | 1000 Genome Phase 3 + UK10K | Logistic regression + inverse-variance weighted meta-analyses with Bonferroni correction | 33 771 858 | <ftp://ftp.ebi.ac.uk/pub/databases/gwas/summary_statistics/ShrineN_30552067_GCST006911/Shrine_30552067_moderate-severe_asthma.txt.gz> |
|  |  |  |  |  |  |  |  |
| **ASD** |  |  |  |  |  |  |  |
| PGC | 23453885 | Childhood ASD | 10 610 | 1000 Genome Phase 3 | Logistic regression + inverse-variance based meta-analysis | 9 499 589 | <https://www.med.unc.edu/pgc/download/asd-gwas-2015/> |
| Grove J et al. | 30804558 | Childhood ASD | 46 351 | 1000 Genome Phase 3 | Logistic regression + inverse variance weighted fixed-effect meta analysis | 9 112 386 | <https://www.med.unc.edu/pgc/download/ipsych-pgc-asd-nov-2017-published-2019/> |
| Massrali A et al. | 31346403 | Autistic trait score at age of 8 yrs | 5 628 | 1000 Genomes Phase 1, Version 3 | Linear regression | 6 977 036 | <ftp://ftp.ebi.ac.uk/pub/databases/gwas/summary_statistics/MassraliA_31346403_GCST008484/scdclogGWAS.Rdata> |

Table S3. Descriptive statistics on the relevant sibling and cousin pairs.

|  | | **Full siblings  (n=1704388 pairs)** | | | | **Maternal half-siblings (n=177202 pairs)** | | | **Paternal half-siblings  (n=179352 pairs)** | | | **Full cousins  (n=3921890 pairs)** | | |  |
| --- | --- | --- | --- | --- | --- | --- | --- | --- | --- | --- | --- | --- | --- | --- | --- |
|  | **All** | | **Female pairs** | **Male pairs** | | **All** | **Female pairs** | **Male pairs** | **All** | **Female pairs** | **Male pairs** | **All** | **Female pairs** | **Male pairs** |  |
| Asthma ever | |  | | |  | | |  | |  | |  |  | |  |
| No | 1478207 (86.73) | | 360235 (88.77) | 386142 (84.93) | 146793 (82.84) | | 35824 (85.15) | 37710 (80.58) | 149993 (83.63) | 36628 (85.80) | 38245 (81.62) | 3330909 (84.93) | 809927 (87.22) | 855662 (82.75) |  |
| Yes (n,%) | 226181 (13.27) | | 45595 (11.23) | 68534 (15.07) | 30409 (17.16) | | 6246 (14.85) | 9086 (19.42) | 29359 (16.37) | 6060 (14.20) | 8611 (18.38) | 590981 (15.07) | 118725 (12.78) | 178388 (17.25) |  |
| Asthma ever > 4.5yr (n,%) | 108353 (6.36) | | 24043 (5.92) | 30270 (6.66) | 65(9.72) | | 2967 (7.05) | 3496 (7.47) | 13179 (7.35) | 3019 (7.07) | 3534 (7.54) | 285502 (7.28) | 63182 (6.80) | 80175 (7.75) |  |
|  | |  | | |  | | |  | |  | |  |  | |  |
| ASD ever | |  | | |  | | |  | |  | |  |  | |  |
| No | 1682379 (98.71) | | 402902 (99.28) | 446423 (98.18) | 172681 (97.45) | | 41407 (98.42) | 45162 (96.51) | 175307 (97.74) | 42112 (98.65) | 45382 (96.85) | 3866790 (98.60) | 920840 (99.16) | 1013995 (98.06) |  |
| Yes (n,%) | 22009 (1.29) | | 2928 (0.72) | 8253 (1.82) | 4521 (2.55) | | 663 (1.58) | 1634 (3.49) | 4045 (2.26) | 576 (1.35) | 1474 (3.15) | 55100 (1.40) | 7812 (0.84) | 20055 (1.94) |  |
| Autistic disorder (n,%) | 9225 (0.54) | | 1088 (0.27) | 3583 (0.79) | 1550 (0.87) | | 208 (0.49) | 600 (1.28) | 1447 (0.81) | 203 (0.48) | 563 (1.20) | 20167 (0.51) | 2578 (0.28) | 7655 (0.74) |  |
| Aspergers syndrome (n,%) | 8220 (0.48) | | 1132 (0.28) | 3005 (0.66) | 1873 (1.06) | | 274 (0.65) | 662 (1.41) | 1661 (0.93) | 217 (0.51) | 572 (1.22) | 23459 (0.60) | 3507 (0.38) | 8407 (0.81) |  |
|  | |  | | |  | | |  | |  | |  |  | |  |
| Having both asthma and ASD (n,%) | 4134 (0.24) | | 482 (0.12) | 1616 (0.36) | 970 (0.55) | | 113 (0.27) | 391 (0.84) | 904 (0.50) | 111 (0.26) | 343 (0.73) | 10890 (0.28) | 1409 (0.15) | 4118 (0.40) |  |

Table S4. Within-individual and their relatives' odds ratios of ASD if exposure to asthma.

| **Relative categories** | |  | Relative  phenotype | OR (95%CI) | | | |
| --- | --- | --- | --- | --- | --- | --- | --- |
|  |  |  |  | Model 1* | Model 2# | Model 3¤ | Model 4§ |
| **Within individual** | | (n=1,569,944) | ASD ever | 1.39 (1.35, 1.44) | 1.33 (1.28, 1.37) | - | - |
|  | |  | Autistic disorder | 1.37 (1.30, 1.44) | 1.26 (1.19, 1.33) | - | - |
|  | |  | Asperger syndrome | 1.41 (1.34, 1.48) | 1.37 (1.30, 1.44) | - | - |
|  | |  |  |  |  |  |  |
| **First degree** | | Full-siblings (n=1,704,388 pairs) | ASD ever | 1.40 (1.35, 1.46) | 1.44 (1.38, 1.50) | 1.35 (1.30, 1.41) | 1.36 (1.31, 1.41) |
|  | |  | Autistic disorder | 1.29 (1.22, 1.38) | 1.32 (1.24, 1.41) | 1.25 (1.18, 1.33) | 1.26 (1.18, 1.34) |
|  | |  | Asperger syndrome | 1.56 (1.47, 1.66) | 1.61 (1.51, 1.71) | 1.50 (1.41, 1.59) | 1.50 (1.41, 1.60) |
|  | |  |  |  |  |  |  |
| **Second degree** | | Maternal half-siblings (n=177,202 pairs) | ASD ever | 1.30 (1.19, 1.40) | 1.28 (1.18, 1.39) | 1.24 (1.15, 1.35) | 1.24 (1.14, 1.34) |
|  | |  | Autistic disorder | 1.21 (1.06, 1.38) | 1.21 (1.06, 1.38) | 1.19 (1.04, 1.36) | 1.18 (1.03, 1.35) |
|  | |  | Asperger syndrome | 1.44 (1.27, 1.62) | 1.40 (1.24, 1.59) | 1.36 (1.20, 1.53) | 1.35 (1.20, 1.53) |
|  | | Paternal half-siblings (n=179,352 pairs) | ASD ever | 1.06 (0.97, 1.16) | 1.05 (0.96, 1.15) | 1.03 (0.94, 1.13) | 1.02 (0.93, 1.12) |
|  | |  | Autistic disorder | 1.05 (0.90, 1.21) | 1.04 (0.90, 1.21) | 1.02 (0.88, 1.18) | 1.02 (0.88, 1.19) |
|  | |  | Asperger syndrome | 1.09 (0.94, 1.25) | 1.07 (0.93, 1.23) | 1.05 (0.91, 1.21) | 1.04 (0.90, 1.20) |
|  | |  |  |  |  |  |  |
| **Third degree** | | Full-cousins (n=3,921,890 pairs) | ASD ever | 1.05 (1.02, 1.08) | 1.06 (1.03, 1.09) | 1.04 (1.02, 1.07) | 1.04 (1.01, 1.07) |
|  | |  | Autistic disorder | 1.02 (0.98, 1.07) | 1.02 (0.98, 1.07) | 1.01 (0.97, 1.06) | 1.01 (0.97, 1.06) |
|  | |  | Asperger syndrome | 1.06 (1.02, 1.11) | 1.07 (1.03, 1.12) | 1.06 (1.02, 1.10) | 1.06 (1.01, 1.10) |
|  |  | |  |  |  |  |  |

*Models 1 did not adjusted for covariates.

#Models 2 adjusted for birth year (categorical), sex and parity (categorical).

¤Models 3 adjusted for birth year (categorical), sex, parity (categorical), and mutually adjusted for the other phenotype (e.g. In order to estimate the OR for ASD ever in the relative, we adjusted for both index person's and relative's asthma).

§Models 4 adjusted for birth year (categorical), sex, parity (categorical), mutually adjusted for the other phenotype (e.g. In order to estimate the OR for ASD ever in the relative, we adjusted for both index person's and relative's asthma), and parental age.

Note: Results to estimate the OR for asthma in the individuals and relatives are rather similar. We decided not to shown here due to the space but they are available upon request.

Table S5. Within-individual and their relatives' odds ratios of ASD if exposure to asthma later than 4.5 years of age.

| **Relative categories** |  | Relative  phenotype | OR (95%CI) | | | |
| --- | --- | --- | --- | --- | --- | --- |
|  |  |  | Model 1* | Model 2# | Model 3¤ | Model 4§ |
| **Within individual** | (n=1,569,944) | ASD ever | 1.33 (1.27, 1.39) | 1.11 (1.06, 1.16) | - | - |
|  |  | Autistic disorder | 1.00 (0.92, 1.08) | 0.93 (0.86, 1.01) | - | - |
|  |  | Asperger syndrome | 1.74 (1.63, 1.85) | 1.27 (1.20, 1.36) | - | - |
|  |  |  |  |  |  |  |
| **First degree** | Full-siblings (n=1,704,388 pairs) | ASD ever | 1.38 (1.31, 1.45) | 1.37 (1.30, 1.44) | 1.35 (1.28, 1.42) | 1.30 (1.23, 1.37) |
|  |  | Autistic disorder | 1.23 (1.13, 1.33) | 1.26 (1.16, 1.37) | 1.27 (1.17, 1.38) | 1.21 (1.16, 1.31) |
|  |  | Asperger syndrome | 1.61 (1.49, 1.73) | 1.51 (1.40, 1.64) | 1.46 (1.35, 1.57) | 1.42 (1.31, 1.53) |
|  |  |  |  |  |  |  |
| **Second degree** | Maternal half-siblings (n=177,202 pairs) | ASD ever | 1.10 (0.98, 1.24) | 1.21 (1.07, 1.36) | 1.20 (1.07, 1.36) | 1.18 (1.05, 1.33) |
|  |  | Autistic disorder | 1.26 (1.04, 1.51) | 1.26 (1.04, 1.52) | 1.26 (1.05, 1.53) | 1.24 (1.03, 1.50) |
|  |  | Asperger syndrome | 1.10 (0.92, 1.32) | 1.31 (1.09, 1.57) | 1.30 (1.08, 1.55) | 1.27 (1.06, 1.53) |
|  | Paternal half-siblings (n=179,352 pairs) | ASD ever | 0.95 (0.84, 1.08) | 1.00 (0.88, 1.14) | 1.00 (0.87, 1.14) | 0.98 (0.86, 1.12) |
|  |  | Autistic disorder | 0.95 (0.77, 1.16) | 0.94 (0.76, 1.15) | 0.93 (0.76, 1.15) | 0.92 (0.75, 1.14) |
|  |  | Asperger syndrome | 0.96 (0.79, 1.18) | 1.08 (0.89, 1.33) | 1.07 (0.88, 1.31) | 1.07 (0.87, 1.30) |
|  |  |  |  |  |  |  |
| **Third degree** | Full-cousins (n=3,921,890 pairs) | ASD ever | 1.08 (1.05, 1.12) | 1.05 (1.01, 1.09) | 1.05 (1.01, 1.09) | 1.04 (1.00, 1.08) |
|  |  | Autistic disorder | 1.02 (0.96, 1.08) | 1.00 (0.94, 1.06) | 1.00 (0.94, 1.06) | 1.00 (0.94, 1.05) |
|  |  | Asperger syndrome | 1.15 (1.09, 1.21) | 1.10 (1.04, 1.16) | 1.10 (1.04, 1.15) | 1.09 (1.03, 1.15) |

*Models 1 did not adjusted for covariates.

#Models 2 adjusted for birth year (categorical), sex and parity (categorical).

¤Models 3 adjusted for birth year (categorical), sex, parity (categorical), and mutually adjusted for the other phenotype (e.g. In order to estimate the OR for ASD ever in the relative, we adjusted for both index person's and relative's asthma).

§Models 4 adjusted for birth year (categorical), sex, parity (categorical), mutually adjusted for the other phenotype (e.g. In order to estimate the OR for ASD ever in the relative, we adjusted for both index person's and relative's asthma), maternal and paternal age at child birth (categorical).

Note: Results to estimate the OR for asthma in the individuals and relatives are rather similar. We decided not to shown here due to the space but they are available upon request.

Table S6. Cross-relative within-trait, cross-trait within-individual, and cross-relative cross-trait correlations for asthma and ASD.

| **Type of relatives** | **No. of concordant pairs^a^** | **No. of double concordant pairs^b^** | **Phenotypic correlation for asthma and ASD ^c^** | **ICC for asthma^d^** | **ICC for ASD** | **CRCT correlation for asthma and ASD^e^** | **Phenotypic correlation for asthma and autisitc disorder^c^** | **ICC for autistic disorder** | **CRCT correlation for asthma and autistic disorder** | **Phenotypic correlation for asthma and Asperger's syndrome^c^** | **ICC for Asperger's syndrome** | **CRCT correlation for asthma and Asperger's syndrome** |
| --- | --- | --- | --- | --- | --- | --- | --- | --- | --- | --- | --- | --- |
| **MZ twins (n= 4,842 pairs)** | | | | | | | | | | | | |
| All | - | - | 0.10  (0.08, 0.12) | 0.89  (0.88, 0.90) | 0.69  (0.68, 0.71) | 0.09  (0.07, 0.11) | 0.10  (0.09, 0.12) | 0.84  (0.83, 0.85) | 0.15  (0.13, 0.17) | -0.18  (-0.20, -0.16) | 0.80  (0.79, 0.81) | -0.18  (-0.20, -0.16) |
| Females | - | - | 0.08  (0.05, 0.10) | 0.89  (0.88, 0.90) | 0.71  (0.69, 0.73) | 0.08  (0.05, 0.11) | 0.12  (0.09, 0.14) | 0.81  (0.80, 0.82) | 0.14  (0.11, 0.17) | -0.25  (-0.27, -0.22) | 0.77  (0.75, 0.79) | -0.25  (-0.27, -0.22) |
| Males | - | - | 0.11  (0.08, 0.14) | 0.89  (0.88, 0.90) | 0.66 (0.63, 0.68) | 0.09  (0.06, 0.12) | 0.07  (0.05, 0.10) | 0.87  (0.86, 0.88) | 0.15  (0.12, 0.18) | -0.04  (-0.07, -0.01) | 0.91  (0.91, 0.92) | -0.04  (-0.07, -0.01) |
|  |  |  |  |  |  |  |  |  |  |  |  |  |
| **DZ twins (n= 11,244 pairs)** | | | | | | | | | | | | |
| All | - | - | 0.13  (0.11, 0.14) | 0.39  (0.38, 0.41) | 0.26  (0.24, 0.28) | 0.08  (0.07, 0.09) | 0.11  (0.09, 0.12) | 0.42  (0.40, 0.43) | 0.08  (0.07, 0.09) | 0.14  (0.13, 0.15) | 0.42  (0.41, 0.44) | 0.13  (0.11, 0.14) |
| Females | - | - | 0.06  (0.03, 0.08) | 0.40  (0.37, 0.43) | 0.26  (0.22, 0.29) | 0.06  (0.04, 0.09) | -0.03  (0.05, 0.00) | 0.50  (0.48, 0.53) | 0.04  (0.01, 0.06) | 0.24  (0.21, 0.26) | 0.51  (0.49, 0.54) | 0.22  (0.20, 0.25) |
| Males | - | - | 0.11  (0.09, 0.14) | 0.47  (0.44, 0.50) | 0.30  (0.27, 0.34) | 0.08  (0.06, 0.11) | 0.15  (0.12, 0.18) | 0.28  (0.25, 0.32) | 0.05  (0.02, 0.08) | -0.02  (-0.05, 0.01) | 0.26  (0.23, 0.30) | 0.04  (0.02, 0.07) |
| Opposite sex | - | - | 0.15  (0.12, 0.18) | 0.34  (0.32, 0.37) | 0.24  (0.22, 0.26) | 0.08  (0.06, 0.10) | 0.17  (0.16, 0.19) | 0.34  (0.31, 0.36) | 0.10  (0.08, 0.12) | 0.09  (0.07, 0.11) | 0.41  (0.39, 0.43) | 0.05  (0.04, 0.07) |
|  |  |  |  |  |  |  |  |  |  |  |  |  |
| **Full-siblings (n=523,893 pairs)** | | | | | | | | | | | | |
| All | 2416 | 68 | 0.08  (0.08, 0.08) | 0.34  (0.33, 0.34) | 0.42  (0.42, 0.43) | 0.06  (0.06, 0.06) | 0.06  (0.06, 0.06) | 0.43  (0.42, 0.43) | 0.04  (0.04, 0.04) | 0.08  (0.07, 0.08) | 0.42  (0.41, 0.42) | 0.07  (0.07, 0.07) |
| Females | 311 | 4 | 0.06  (0.06, 0.07) | 0.36  (0.36, 0.37) | 0.43  (0.43, 0.44) | 0.06  (0.06, 0.07) | 0.04  (0.03, 0.04) | 0.47  (0.46, 0.47) | 0.05  (0.05, 0.06) | 0.07  (0.07, 0.07) | 0.39  (0.39, 0.40) | 0.08  (0.07, 0.08) |
| Males | 990 | 32 | 0.08  (0.07, 0.08) | 0.34  (0.34, 0.35) | 0.43  (0.43, 0.44) | 0.08  (0.08, 0.09) | 0.06  (0.05, 0.06) | 0.41  (0.41, 0.42) | 0.06  (0.05, 0.06) | 0.07  (0.06, 0.07) | 0.47  (0.47, 0.48) | 0.07  (0.07, 0.08) |
| Opposite sex | 1115 | 32 | 0.08  (0.08, 0.08) | 0.31  (0.31, 0.31) | 0.40  (0.39, 0.40) | 0.04  (0.04, 0.04) | 0.07  (0.07, 0,07) | 0.37  (0.37, 0.37) | 0.02  (0.01, 0.02) | 0.07  (0.07, 0.08) | 0.41  (0.40, 0.41) | 0.05  (0.05, 0.06) |
|  |  |  |  |  |  |  |  |  |  |  |  |  |
| **Maternal half-siblings (n=50,303 pairs)** | | | | | | | | | | | | |
| All | 497 | 11 | 0.08  (0.07, 0.08) | 0.21  (0.21, 0.22) | 0.24  (0.23, 0.25) | 0.06  (0.05, 0.06) | 0.06  (0.06, 0.07) | 0.19  (0.18, 0.20) | 0.04  (0.03, 0.04) | 0.07  (0.06, 0.08) | 0.19  (0.18, 0.19) | 0.07  (0.07, 0.08) |
| Females | 71 | 2 | 0.08  (0.06, 0.09) | 0.21  (0.20, 0.23) | 0.29  (0.28, 0.31) | 0.05 (0.04, 0.06) | 0.05  (0.04, 0.06) | 0.28  (0.26, 0.30) | 0.03  (0.01, 0.04) | 0.07  (0.05, 0.08) | 0.21  (0.20, 0.23) | 0.06  (0.05, 0.08) |
| Males | 191 | 7 | 0.05  (0.04, 0.06) | 0.21  (0.19, 0.22) | 0.19  (0.17, 0.21) | 0.08  (0.06, 0.09) | 0.06  (0.04, 0.07) | 0.10  (0.08, 0.12) | 0.08  (0.07, 0.09) | 0.08  (0.06, 0.09) | 0.09  (0.07, 0.11) | 0.07  (0.06, 0.08) |
| Opposite sex | 235 | 2 | 0.07  (0.06, 0.08) | 0.21  (0.20, 0.22) | 0.18  (0.17, 0.20) | 0.04  (0.04, 0.05) | 0.06  (0.05, 0.07) | 0.05  (0.03, 0.06) | 0.02  (0.01, 0.03) | 0.06  (0.05, 0.07) | 0.22  (0.21, 0.23) | 0.07  (0.06, 0.08) |
|  |  |  |  |  |  |  |  |  |  |  |  |  |
| **Paternal half-siblings (n=46,798 pairs)** | | | | | | | | | | | | |
| All | 416 | 3 | 0.10  (0.09, 0.10) | 0.12  (0.11, 0.13) | 0.18  (0.17, 0.19) | 0.02  (0.01, 0.02) | 0.09  (0.09, 0.10) | 0.24  (0.24, 0.25) | 0.03  (0.02, 0.03) | 0.08  (0.08, 0.09) | 0.10  (0.09, 0.11) | 0.01  (0.01, 0.02) |
| Females | 47 | 0 | 0.11  (0.09, 0.12) | 0.13  (0.11, 0.14) | 0.21  (0.20, 0.23) | 0.00  (-0.01, 0.02) | 0.08  (0.07, 0.10) | 0.21  (0.19, 0.23) | 0.03  (0.01, 0.04) | 0.08  (0.07, 0.10) | 0.09  (0.07, 0.10) | -0.01  (-0.03, 0.00) |
| Males | 195 | 1 | 0.08  (0.07, 0.09) | 0.10  (0.09, 0.12) | 0.18  (0.16, 0.20) | 0.01  (0.00, 0.03) | 0.03  (0.02, 0.04) | 0.33  (0.32, 0.35) | -0.13  (-0.14, -0.12) | 0.11  (0.09, 0.12) | 0.27  (0.26, 0.29) | 0.07  (0.05, 0.08) |
| Opposite sex | 174 | 2 | 0.08  (0.07, 0.09) | 0.11  (0.10, 0.12) | 0.11  (0.10, 0.13) | 0.02  (0.01, 0.03) | 0.10  (0.09, 0.11) | 0.22  (0.21, 0.24) | 0.04  (0.03, 0.05) | 0.06  (0.05, 0.07) | 0.04  (-0.03, 0.05) | 0.00  (-0.01, 0.01) |

**Note**:

a. Number of concordant pairs denotes number of sibling pairs where one sibling had asthma and the other sibling had ASD. For twins, parent-reported asthma and ASD trait was measured as scores, so concordant pairs not applicable.

b. Number of double concordant pairs denotes sibling pairs where both siblings had asthma and ASD.

c. Phenotypic correlation denotes the correlation coefficient between asthma and ASD within individual for non-twins.

d. Intraclass correlation (ICC) denotes the correlation between individual and his/her relative on the trait. e. Cross-relative cross-trait (CRCT) correlation is presented as the tetrachoric correlation when the individual had asthma, and the relative of the individual had ASD.

e. Cross-relative cross-trait correlation (CRCT) denotes the tetrachoric correlation when the individual had asthma ever, and the relative of the individual had ASD ever, autistic disorder, or Asperger's syndrome. For twins, we used the parent-reported asthma and ASD trait measures instead of the asthma and ASD ever and reported the biserial correlation coefficients with confidence intervals.

**Abbreviations**: ICC-Intraclass correlation; CRCT correlation - Cross relative cross trait correlation

Table S7. Cross-sibling within-trait, cross-trait within-sibling, and cross-sibling cross-trait correlations for asthma 4.5yr+ and ASD.

| Zygosity | No. of concordant pairs^a^ | No. of double concordant pairs^b^ | Phenotypic correlation^c^ | ICC for asthma 4.5yr+ ^d^ | ICC for ASD ever | CSCT correlation for asthma 4.5yr+ and ASD ever^e^ |
| --- | --- | --- | --- | --- | --- | --- |
| Full-siblings (n=523,893 pairs) | | | | | | |
| All | 1236 | 21 | 0.06 (0.06, 0.06) | 0.26 (0.26, 0.27) | 0.42 (0.42, 0.43) | 0.06 (0.06, 0.06) |
| Females | 187 | 1 | 0.06 (0.05, 0.06) | 0.27 (0.27, 0.28) | 0.43 (0.43, 0.44) | 0.06 (0.05, 0.06) |
| Males | 457 | 10 | 0.07 (0.07, 0.08) | 0.29 (0.28, 0.29) | 0.43 (0.43, 0.44) | 0.09 (0.09, 0.10) |
| Opposite sex | 592 | 10 | 0.06 (0.06, 0.06) | 0.25 (0.25, 0.25) | 0.40 (0.39, 0.40) | 0.05 (0.05, 0.05) |
| Maternal half-siblings (n=50,303 pairs) | | | | | | |
| All | 184 | 4 | 0.07 (0.06, 0.07) | 0.10 (0.09, 0.11) | 0.24 (0.23, 0.25) | 0.00 (0.00, 0.01) |
| Females | 35 | 2 | 0.07 (0.06, 0.09) | 0.08 (0.06, 0.10) | 0.29 (0.28, 0.31) | -0.01 (-0.02, 0.00) |
| Males | 63 | 2 | 0.06 (0.05, 0.07) | 0.14 (0.13, 0.16) | 0.19 (0.17, 0.21) | 0.06 (0.05, 0.07) |
| Opposite sex | 86 | 0 | 0.06 (0.05, 0.07) | 0.09 (0.08, 0.11) | 0.18 (0.17, 0.20) | -0.01 (-0.02, 0.00) |
| Paternal half-siblings (n=46,798 pairs) | | | | | | |
| All | 159 | 0 | 0.08 (0.08, 0.09) | 0.04 (0.03, 0.04) | 0.18 (0.17, 0.19) | 0.01 (0.00, 0.01) |
| Females | 28 | 0 | 0.09 (0.07, 0.10) | 0.04 (0.02, 0.06) | 0.21 (0.20, 0.23) | -0.05 (-0.06, -0.03) |
| Males | 49 | 0 | 0.07 (0.05, 0.08) | 0.02 (0.01, 0.04) | 0.18 (0.16, 0.20) | 0.05 (0.04. 0.06) |
| Opposite sex | 82 | 0 | 0.08 (0.07, 0.09) | 0.04 (0.03, 0.05) | 0.11 (0.10, 0.13) | 0.03 (0.02, 0.04) |

Note:

a. Number of concordant pairs denotes number of sibling pairs where one sibling had asthma (measured as asthma 4.5yr+) and the other sibling had ASD ever.

b. Number of double concordant pairs denotes sibling pairs where both siblings had asthma 4.5yr+ and ASD ever.

c. Phenotypic correlation denotes the correlation coefficient between asthma 4.5yr+ and ASD ever within individual for non-twins.

d. Intraclass correlation (ICC) denotes the correlation between individual and his/her relative on the trait.

e. Cross-relative cross-trait correlation (CRCT) denotes the tetrachoric correlation when the individual had asthma 4.5yr+, and the relative of the individual had ASD ever.

Table S8. Quantitative genetic modelling - Bivariate Cholesky model. Genetic and environmental parameter estimates for familial co-aggregation of asthma and ASD using 15963 pairs of twins.

|  | Models | | |
| --- | --- | --- | --- |
| Parameters | ACE | ADE | AE |
| *Asthma, % (95% CI)* |  |  |  |
| Asthma due to A | 0.67 (0.67, 0.68) | 0.65 (0.50, 0.79) | 0.88 (0.86, 0.90) |
| Asthma due to D | na | 0.24 (0.09, 0.39) | na |
| Asthma due to H | na | 0.89 (0.87, 0.91) | na |
| Asthma due to C | 0 | na | na |
| Asthma due to E | 0.12 (na, na) | 0.11 (0.09, 0.13) | 0.12 (0.10, 0.14) |
| *ASD trait, % (95% CI)* |  |  |  |
| ASD trait due to A | 0.69 (na, na) | 0.13 (0.10, 0.16) | 0.43 (0.42, 0.44) |
| ASD trait due to D | na | 0.37 (0.33, 0.41) | na |
| ASD trait due to H | na | 0.50 (0.49, 0.51) | na |
| ASD trait due to C | 0 | na | na |
| ASD trait due to E | 0.31 (na, na) | 0.50 (0.49, 0.51) | 0.57 (0.56, 0.58) |
| *Bivariate explained covariance, % (95% CI)* |  |  |  |
| Covariance due to A | 1.04 (0.92, na) | 1.53 (0.77, 2.30) | 1.00 (0.81, 1.21) |
| Covariance due to D | na | -0.65 (-1.49, 0.20) | na |
| Covariance due to H | na | 0.89 (0.68, 1.10) | na |
| Covariance due to C | 0 | na | na |
| Covariance due to E | -0.04 (-0.16, 0.09) | 0.11 (-0.10, 0.32) | 0.00 (-0.21, 0.20) |
| *Bivariate correlations, % (95% CI)* |  |  |  |
| rA | 0.15 (na, na) | 0.31 (0.15, 0.47) | 0.10 (0.07, 0.12) |
| rD | na | -0.13 (-0.30, 0.05) | na |
| rH | na | 0.20 (0.14, 0.26) | na |
| rC | 1.00 (na, na) | na | na |
| rE | -0.02 (na, na) | 0.03 (-0.02, 0.08) | 0.00 (-0.05, 0.05) |

Notes:

1. After running the saturated model and several assumption models, we used the base model with equal threshold to build the ACE, ADE, AE models. We used a weighted least squares approach to model the point estimate and the 95% confidence intervals (CI) based on standard errors (Wald CI). Therefore, the 95% CIs might fall above 1 or below -1 for correlations, and below 0 for variance components. The best fitting model – ADE – includes additive and non-additive/dominant genetic and non-shared environment parameters (with lowest AIC).

2. All models were adjusted for sex and birth year (continuous, standardized).

Abbreviations: A, additive genetic component; D, non-additive/ dominant genetic component; H, broad-sense heritability component, which is A+D; C, shared environmental component; E, non-shared environmental component (including measurement errors); rA, additive genetic correlation; rD dominant genetic correlation; rH, correlation atrributable to broad-sense heritability; rC, shared environmental correlation; rE, non-shared environmental correlation; na, not applicable.

Table S9. Quantitative genetic modelling- Bivariate Cholesky model. Genetic and environmental parameter estimates for familial co-aggregation of asthma and ASD using 620994 pairs of siblings.

|  | Models | |
| --- | --- | --- |
| Parameters | ACE | AE |
| *Asthma, % (95% CI)* |  |  |
| Asthma due to A | 0.46 (0.40, 0.52) | 0.67 (0.66, 0.68) |
| Asthma due to C | 0.10 (0.08, 0.13) | na |
| Asthma due to E | 0.43 (0.40, 0.46) | 0.33 (0.32, 0.34) |
| *ASD trait, % (95% CI)* |  |  |
| ASD trait due to A | 0.76 (0.68, 0.84) | 0.83 (0.80, 0.85) |
| ASD trait due to C | 0.03 (0.00, 0.07) | na |
| ASD trait due to E | 0.21 (0.16, 0.26) | 0.17 (0.15, 0.20) |
| *Bivariate explained covariance, % (95% CI)* |  |  |
| Covariance due to A | 1.49 (0.88, 2.09) | 1.48 (1.31, 1.65) |
| Covariance due to C | 0.00 (-0.24, 0.23) | na |
| Covariance due to E | -0.49 (-0.88, -0.08) | -0.48 (-0.65, -0.31) |
| *Bivariate correlations, % (95% CI)* |  |  |
| rA | 0.20 (0.11, 0.29) | 0.16 (0.15, 0.17) |
| rC | 0.00 (-0.32, 0.31) | na |
| rE | -0.13 (-0.21, -0.04) | -0.16 (-0.20, -0.12) |

Notes:

1. After running the saturated model and several assumption models, we used the base model with equal threshold to build the ACE and AE models. As D components are minimal in half-siblings, we did not try to fit ADE models here.

2. All models were adjusted for sex and birth year (continuous, standardized).

Abbreviations: A, additive genetic parameter; D, non-additive/ dominant genetic parameter; C, shared environmental parameter; E, non-shared environmental parameter (including measurement errors); rA, additive genetic correlation; rD dominant genetic correlation; rC, shared environmental correlation; rE, non-shared environmental correlation; na, not applicable.

Table S10. SNP-based heritability (h^2^_SNP_) and genetic correlation (r_g_) estimates of asthma and ASD.

|  | **N (n_case_: n_control_)** | **Nr. of SNPs remained in the analysis** | **SNP-heritability, h^2^_SNP_ (SE) ^1^** | **Intercept (SE) ^2^** | **Ratio (SE) ^3^** |
| --- | --- | --- | --- | --- | --- |
| ***Univariate analyses*** |  |  |  |  |  |
| Childhood asthma (Moffatt MF et al.) | 26475 (10365:16110) | 514719 | 0.1073 (0.024) | 1.0159 (0.0074) | 0.2398 (0.1116) |
| Childhood asthma (Demenais F et al.) | 127669 (19954:107715) | 1041668 | 0.0541 (0.0072) | 1.0564 (0.009) | 0.2912 (0.0464) |
| Childhood asthma (Ferreira MA et al.) | 314633 (13962:300671) | 1168396 | 0.0591 (0.0061) | 1.0644 (0.0114) | 0.1446 (0.0256) |
| Self-reported asthma (UKB) | 361141 (41934:319207) | 1078769 | 0.0646 (0.0084) | 1.002 (0.0073) | 0.1182 (0.0256) |
| Doctor-diagnosed asthma (UKB) | 91787 (11717:80070) | 1078769 | 0.0528 (0.0043) | 1.0521 (0.0113) | 0.0166 (0.0612) |
| Moderate-to-severe asthma (Shrine N et al.) | 57695 (10549:47416) | 1007069 | 0.1 (0.0113) | 0.9996 (0.0073) | < 0 |
| ASD (PGC) | 10610 (5305:5305) | 1180242 | 0.4618 (0.0523) | 0.9638 (0.0067) | < 0 |
| ASD (Grove J et al.) | 46350 (18381:27969) | 1155641 | 0.203 (0.0154) | 0.9992 (0.0079) | < 0 |
| Autistic trait (Massrali A et al.) | 5628 (at continuous scale) | 1058989 | 0.0993 (0.0834) | 1.0006 (0.0065) | 0.0518 (0.5323) |
|  |  |  |  |  |  |
| ***Bivariate analyses*** | **h^2^ _SNP (trait 1):_ h^2^ _SNP (trait 2)_** | **Genetic correlation, r_g_(SE)** | **p-value** | **Intercept _(trait 1):_ Intercept _(trait 2)_** | **Ratio _(trait 1):_ Ratio _(trait 2)_** |
| Childhood asthma (Moffatt MF et al.) - ASD (PGC) | 0.1161:0.4247 | 0.116 (0.1095) | 0.2897 | 1.0112:0.9739 | 0.171:< 0 |
| Childhood asthma (Moffatt MF et al.) - ASD (Grove J et al.) | 0.1255:0.2089 | -0.0948 (0.0826) | 0.2513 | 1.0061:0.9962 | 0.0939:<0 |
| Childhood asthma (Moffatt MF et al.) - Autistic trait (Massrali A et al.) | 0.1252:0.1224 | 0.063 (0.261) | 0.8094 | 1.007:0.9985 | 0.1053:<0 |
|  |  |  |  |  |  |
| Childhood asthma (Demenais F et al.) - ASD (PGC) | 0.0592:0.4268 | 0.1072 (0.079) | 0.1749 | 1.0448:0.9715 | 0.2314:<0 |
| Childhood asthma (Demenais F et al.) - ASD (Grove J et al.) | 0.0586:0.2031 | 0.0073 (0.0611) | 0.9052 | 1.0461:0.9973 | 0.2372:<0 |
| Childhood asthma (Demenais F et al.) - Autistic trait (Massrali A et al.) | 0.0587:0.1461 | 0.0205 (0.1564) | 0.1309 | 1.047:0.9956 | 0.2375:<0 |
|  |  |  |  |  |  |
| Childhood asthma (Ferreira MA et al.) - ASD (PGC) | 0.0654:0.451 | 0.0072 (0.0465) | 0.8775 | 1.0332:0.967 | 0.0746:<0 |
| Childhood asthma (Ferreira MA et al.) - ASD (Grove J et al.) | 0.0659:0.2023 | 0.0338 (0.0422) | 0.4232 | 1.031:0.997 | 0.069:<0 |
| Childhood asthma (Ferreira MA et al.) - Autistic trait (Massrali A et al.) | 0.0671:0.0986 | 0.1769 (0.1441) | 0.2196 | 1.0159:1.0005 | 0.0348:0.0442 |
|  |  |  |  |  |  |
| Self-reported asthma (UKB) - ASD (PGC) | 0.0569:0.455 | -0.0038 (0.0498) | 0.9387 | 1.0283:0.9661 | 0.0642:<0 |
| Self-reported asthma (UKB) - ASD (Grove J et al.) | 0.0571:0.2022 | 0.0826 (0.0427) | 0.0528 | 1.0278:1.0007 | 0.0627:0.0038 |
| Self-reported asthma (UKB) - Autistic trait (Massrali A et al.) | 0.0579:0.0997 | -0.0272 (0.1247) | 0.8273 | 1.0168:1.0013 | 0.037:0.0981 |
|  |  |  |  |  |  |
| Doctor-diagnosed asthma (UKB) - ASD (PGC) | 0.0675:0.455 | -0.0264 (0.0719) | 0.7139 | 0.9972:0.9661 | <0:<0 |
| Doctor-diagnosed asthma (UKB) - ASD (Grove J et al.) | 0.0683:0.2022 | 0.0219 (0.0585) | 0.7087 | 0.9958:1.0007 | <0:0.0038 |
| Doctor-diagnosed asthma (UKB) - Autistic trait (Massrali A et al.) | 0.07:0.0997 | -0.0899 (0.1723) | 0.6018 | 0.992:1.0013 | <0:0.0981 |
|  |  |  |  |  |  |
| Moderate-to-severe asthma (Shrine N et al.) - ASD (PGC) | 0.1017:0.4662 | 0.0484 (0.0674) | 0.4723 | 0.998:0.9638 | <0:<0 |
| Moderate-to-severe asthma (Shrine N et al.) - ASD (Grove J et al.) | 0.1017:0.2018 | 0.0352 (0.0559) | 0.5285 | 0.9985:0.999 | <0:<0 |
| Moderate-to-severe asthma (Shrine N et al.) - Autistic trait (Massrali A et al.) | 0.1039:0.1058 | -0.0542 (0.1906) | 0.7761 | 0.9935:0.9995 | <0:<0 |

**Note:**

1. SNP heritability - the proportion of variance in the phenotype explained by common genetic variants.

2. Intercept is the LD Score regression intercept. The intercept should be close to 1, unless the data have been genomic control corrected, in which case it will often be lower.

3. Ratio is (intercept-1)/(mean(chi^2)-1), which measures the proportion of the inflation in the mean chi^2 that the LD Score regression intercept ascribes to causes other than polygenic heritability. When the ratio is less than zero, it usually indicates genomic control correlation.

**Abbreviations:**

LD - linkage disequilibrium, PGC - psychiatric genomic consortium, SE - standard error, UKB - UK Biobank

**Reference**

Ekbom, A. (2011). The Swedish Multi-generation Register. *Methods in Molecular Biology, 675*, 215-220. doi:10.1007/978-1-59745-423-0_10

Johansson, L. A., & Westerling, R. (2000). Comparing Swedish hospital discharge records with death certificates: implications for mortality statistics. *International Journal of Epidemiology, 29*(3), 495-502. doi:10.1093/ije/29.3.495

Ludvigsson, J. F., Andersson, E., Ekbom, A., Feychting, M., Kim, J. L., Reuterwall, C., . . . Olausson, P. O. (2011). External review and validation of the Swedish national inpatient register. *BMC Public Health, 11*, 450. doi:10.1186/1471-2458-11-450

Ludvigsson, J. F., Otterblad-Olausson, P., Pettersson, B. U., & Ekbom, A. (2009). The Swedish personal identity number: possibilities and pitfalls in healthcare and medical research. *European Journal of Epidemiology, 24*(11), 659-667. doi:10.1007/s10654-009-9350-y

Magnusson, P. K., Almqvist, C., Rahman, I., Ganna, A., Viktorin, A., Walum, H., . . . Lichtenstein, P. (2013). The Swedish Twin Registry: establishment of a biobank and other recent developments. *Twin Research and Human Genetics, 16*(1), 317-329. doi:10.1017/thg.2012.104

Ortqvist, A. K., Lundholm, C., Wettermark, B., Ludvigsson, J. F., Ye, W., & Almqvist, C. (2013). Validation of asthma and eczema in population-based Swedish drug and patient registers. *Pharmacoepidemiology and Drug Safety, 22*(8), 850-860. doi:10.1002/pds.3465

Swedish National Board of Health and Welfare. (2003). The Swedish Medical Birth Register: a summary of content and quality. Retrieved from <https://www.socialstyrelsen.se/globalassets/sharepoint-dokument/artikelkatalog/ovrigt/2003-112-3_20031123.pdf>

Wettermark, B., Hammar, N., Fored, C. M., Leimanis, A., Otterblad Olausson, P., Bergman, U., . . . Rosen, M. (2007). The new Swedish Prescribed Drug Register--opportunities for pharmacoepidemiological research and experience from the first six months. *Pharmacoepidemiology and Drug Safety, 16*(7), 726-735. doi:10.1002/pds.1294
